# Schwannoma arising in mediastinal lymph node diagnosed by endobronchial ultrasound

**DOI:** 10.1002/rcr2.481

**Published:** 2019-08-20

**Authors:** Lae Hyung Kang, Dong Hoon Shin, Seong‐Hoon Yoon

**Affiliations:** ^1^ Department of Internal Medicine, School of Medicine Pusan National University Yangsan Republic of Korea; ^2^ Department of Pathology, School of Medicine Pusan National University Yangsan Republic of Korea

**Keywords:** Schwannoma, lymph node, endobronchial ultrasound

## Abstract

Schwannoma is a peripheral nerve sheath tumour that largely originates from the posterior mediastinum. Schwannoma arising in mediastinal lymph node is extremely rare. A 53‐year‐old female was referred to our hospital for the evaluation of enlarged mediastinal lymph node. Computed tomography scan revealed a non‐enhancing soft tissue mass at Rt. paratracheal area. Endobronchial ultrasound showed a well‐defined round‐shaped lymph node and transbronchial needle aspiration was performed from this lymph node. Pathologic findings revealed spindle cells with strong S100 positivity. Complete surgical excision was done. We report a rare case of schwannoma arising in mediastinal lymph node diagnosed by endobronchial ultrasound.

## Introduction

Schwannoma is a tumour that originates from peripheral nerve sheath. It is generally located in the posterior mediastinum. Schwannoma arising in mediastinal lymph node is extremely rare. The diagnosis is usually based on pathologic examination via surgical excision. We present a case of intranodal schwannoma diagnosed by endobronchial ultrasound‐guided transbronchial needle aspiration (EBUS‐TBNA).

## Case Report

**Figure 1:**
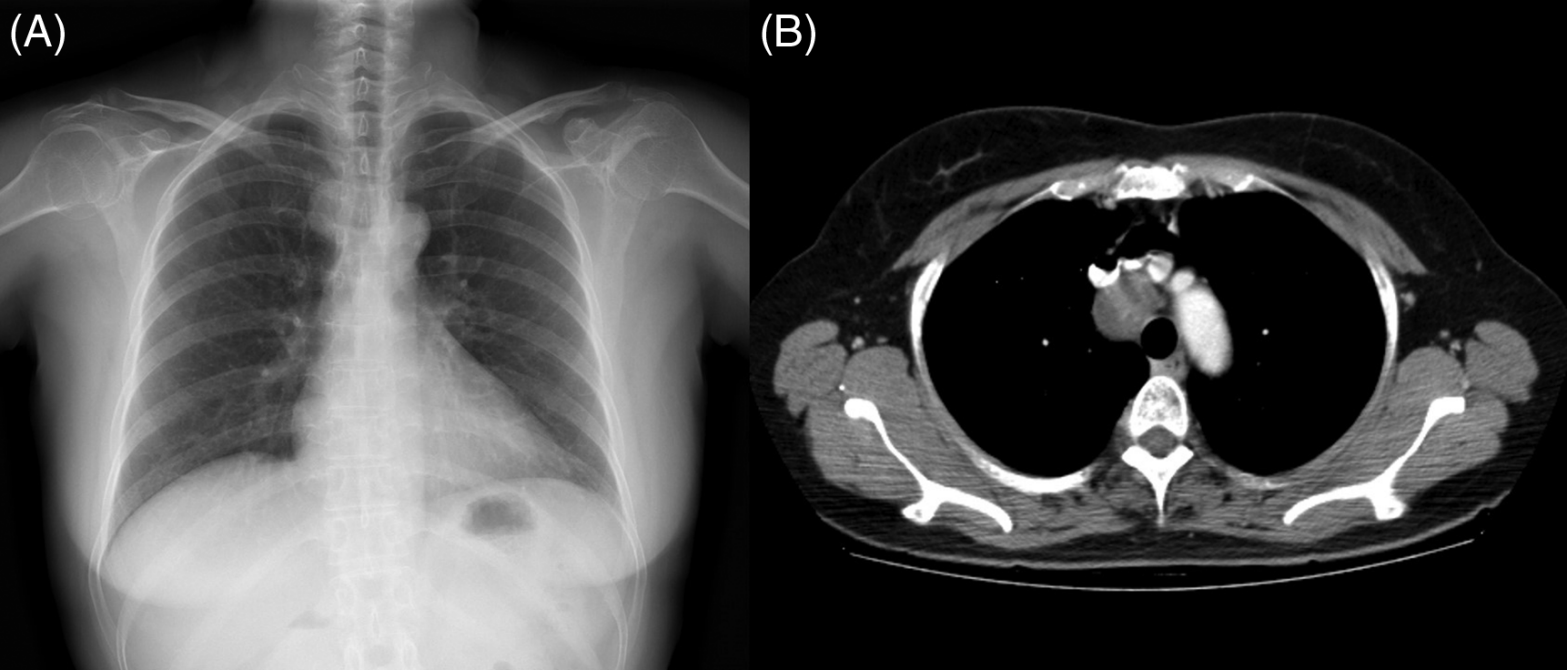
Chest imaging. (A) Chest X‐ray shows right mediastinal enlargement. (B) Computed tomography of chest reveals a soft tissue density in the right paratracheal area.

**Figure 2:**
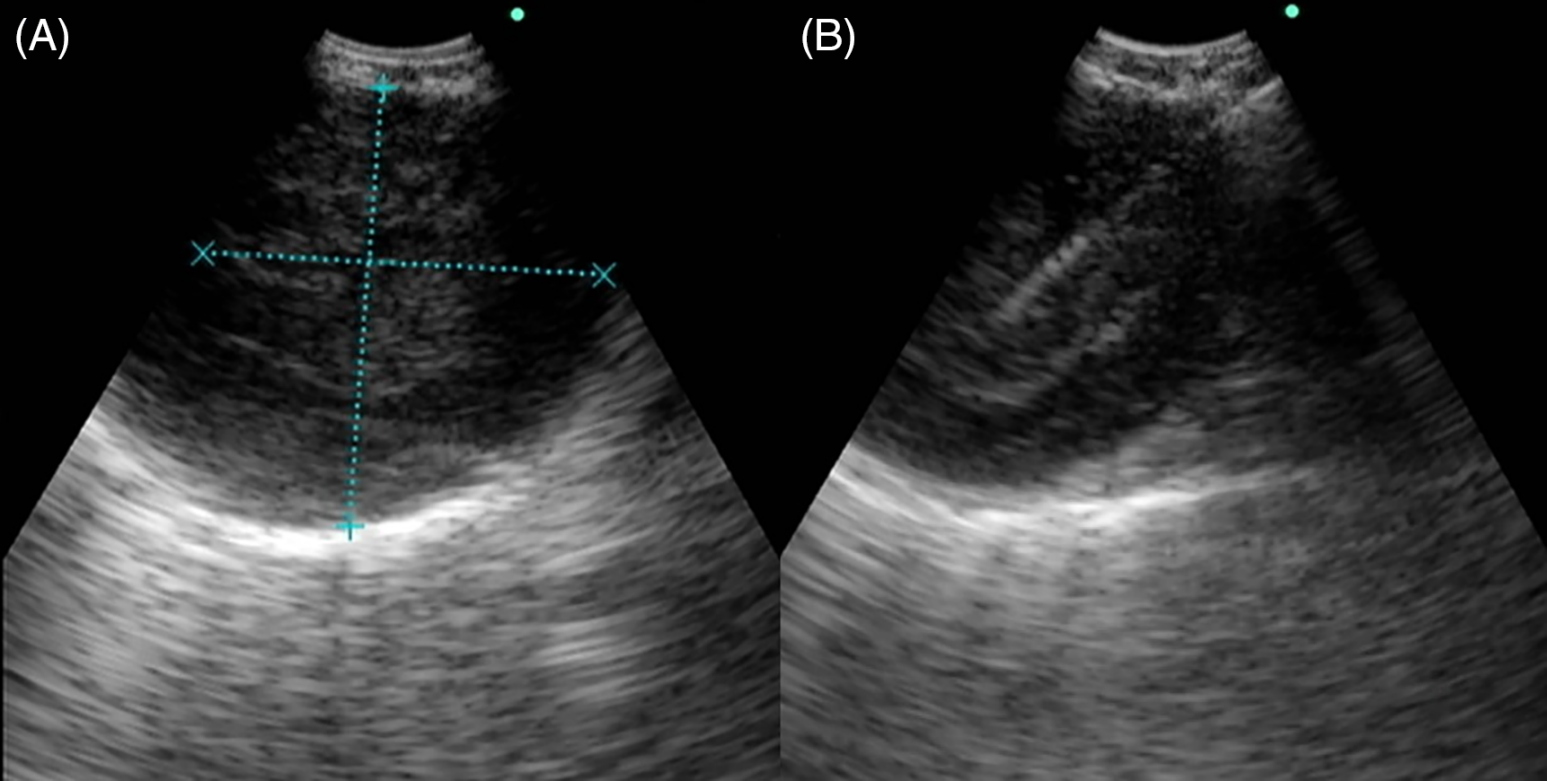
Endobronchial ultrasound (EBUS). (A, B) EBUS shows a well‐defined hypoechoic mass with posterior acoustic enhancement.

**Figure 3:**
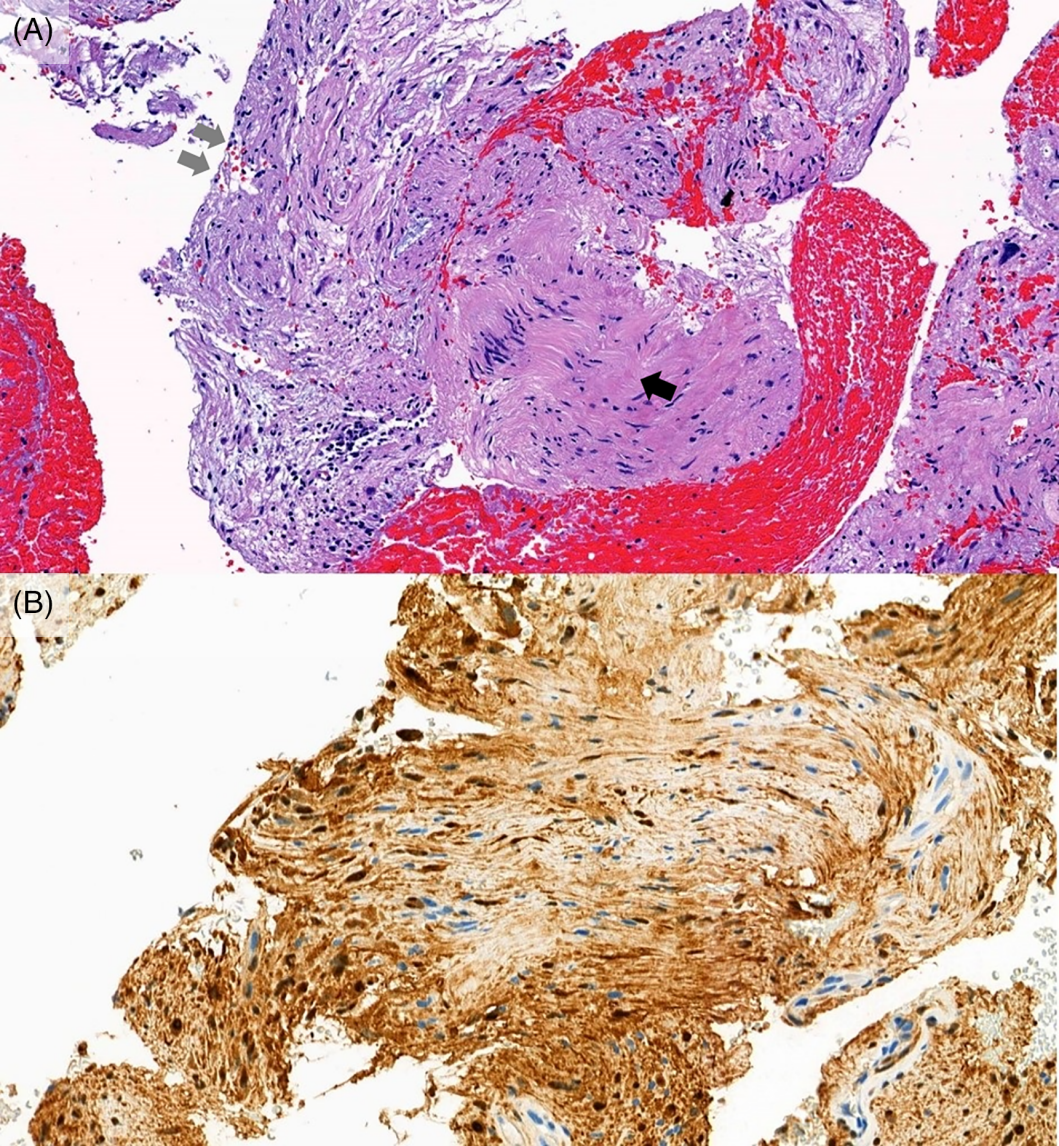
Pathologic images. (A) The sample obtained by endobronchial ultrasound‐guided transbronchial needle aspiration from mediastinal lymph node shows a schwannoma composed of spindle mesenchymal cells of benign feature forming irregular bundles (haematoxylin and eosin staining, ×100). The grey arrow and black arrow indicate Antoni A and Antoni B respectively (B) Immunohistochemical staining shows diffuse and strong positivity for S‐100 (×200).

A 53‐year‐old female was referred to our hospital for the further evaluation of enlarged mediastinal lymph node, which was incidentally found on computed tomography (CT) scan. She was a non‐smoker and had no past medical history. Her physical examinations showed a slender woman who did not have ill appearance with stable vital sign. Chest X‐ray showed a mediastinal mass in the upper zone of right lung (Fig. 1). Computed tomography chest revealed a non‐enhancing soft tissue lesion with a size of approximately 3.0 × 2.4 cm in the right lower paratracheal region (Fig.1). Endobronchial ultrasound showed a well‐defined, round‐shaped lymph node with heterogenous echogenic nature at the right paratracheal area (Fig. 2). Endobronchial ultrasound‐guided transbronchial needle aspiration was performed from this area and enough tissue cores were obtained. Histopathologic findings showed a benign looking spindle cell proliferation with diffuse and strong positivity for S‐100 in immunohistochemical staining, which was compatible with schwannoma (Fig. 3). She underwent mass excision via video‐assisted thoracoscopic surgery (VATS). Grossly the excised mass was a well‐encapsulated tissue that measured 3.4 × 3.3 × 2.7 cm in dimensions. On section, the cut surface was gelatinous and yellow‐white coloured. Microscopic findings showed a spindle cell lesion and mucinous stoma with variable amount of collagen. Compressed lymphoid tissue was seen at the periphery with positivity for leukocyte common antigen. In immunohistochemical staining, vimentin and S‐100 were strongly positive, but smooth muscle actin (SMA), human melanoma black (HMB)‐45, and cytokeratin (CK) were all negative.

## Discussion

Schwannoma is a subtype of neurogenic tumour, which accounts for 21% of all primary mediastinal tumours in adults 1. It is a benign tumour that originates from schwann cells derived from neural crest and has slowly growing feature with low malignant potential 2. It is generally solitary and more common in women 3. Mediastinal schwannoma is most commonly originated in costovertebral angles in the posterior mediastinum 4. It is usually asymptomatic and is often incidentally found radiographically.

Intranodal schwannoma is extremely rare 5. On CT chest, schwannoma shows well‐defined heterogenous attenuation with low attenuation area representing hypocellularity or cystic degeneration 6. Positron emission tomography (PET)‐CT has additional advantage of checking the metabolic activity of mass to differentiate between benign and malignant lesions. Especially, it is important to distinguish intranodal schwannoma from lymph node metastasis. However, PET‐CT appears to be ineffective for distinguishing schwannoma from malignancy because of variable fluorodeoxyglucose (FDG) uptake in schwannoma 7. Magnetic resonance imaging (MRI) can be useful for the diagnosis of intranodal schwannoma. On T1‐weighted images, mass appears to be homogenous and isointense relative to skeletal muscle, while T2‐weighted images show increased, and heterogenous intensity 8. However, it is rather difficult to give a precise diagnosis using imaging studies.

Schwannoma is usually diagnosed by histologic examination. Biopsy for intranodal schwannoma can be performed via surgery like mediastinoscopy, EBUS‐TBNA or endoscopic ultrasound‐guided fine needle aspiration (EUS‐FNA). In this case we could obtain tissue samples from the lymph node by using EBUS‐TBNA. There are only a few cases of schwannoma diagnosed by EBUS‐TBNA in the literature 9. On ultrasound, schwannoma has a well‐defined hypoechogenic feature. An echogenic capsule, and posterior acoustic enhancement can be also seen in some cases 10.

Microscopic appearance of schwannoma is characterized by mixture of two distinct patterns, Antoni A and B. More cellular areas (Antoni A) are composed of spindle cells, while Antoni B areas are less cellular in connective tissue stroma. In immunohistrochemical staining, schwannoma shows the positivity for S‐100 strongly 11. We performed several other staining like SMA, HMB‐45, and CK to rule out intranodal myofibroblastoma, spindle cell carcinoma, and spindle cell melanoma. Schwannoma is generally a benign tumour, but there are a few cases showing malignant transformation reported in the literature 12. Surgical resection is recommended as the definitive treatment for schwannoma, based on local mass effect and potential for malignant transformation.

In conclusion, we report a rare case of schwannoma arising in mediastinal lymph node diagnosed by EBUS‐TBNA. This procedure might be helpful for the diagnosis of intranodal schwannoma.

## Disclosure statement

Appropriate written informed consent was obtained for publication of this case report and accompanying images.
